# Quadratic Non-Linear Optical Properties of the poly(2,5-*bis*(but-2-ynyloxy) Benzoate Containing the 2-(ethyl(4-((4-nitrophenyl)buta-1,3-diynyl)phenyl)amino)ethanol) Chromophore

**DOI:** 10.3390/polym12010241

**Published:** 2020-01-20

**Authors:** Sandra L. Castañón-Alonso, Omar G. Morales-Saavedra, Marco A. Almaraz-Girón, Sandro Báez-Pimiento, Alejandro Islas-Jácome, L. M. Rocha-Ramírez, Armando Domínguez-Ortiz, Marcos Esparza-Schulz, Adolfo Romero-Galarza, María E. Hernández-Rojas

**Affiliations:** 1 Departamento de Química, Universidad Autónoma Metropolitana-Iztapalapa, San Rafael Atlixco 186, Col. Vicentina, Iztapalapa, Ciudad de México C.P. 09340, Mexico; aij@xanum.uam.mx (A.I.-J.); doar@xanum.uam.mx (A.D.-O.); esma@xanum.uam.mx (M.E.-S.); 2 Instituto de Ciencias Aplicadas y Tecnología, Universidad Nacional Autónoma de México, Circuito Exterior S/N, Ciudad Universitaria, Coyoacán, Ciudad de México C.P. 04510, Mexico; omar.morales@ccadet.unam.mx; 3 Departamento de Ingeniería Industrial, Facultad de Ingeniería y Arquitectura, Universidad Nacional de Colombia, Sede Manizales, Km. 7 vía al Aeropuerto, Manizales C.P. 4-72, Colombia; sbaezp@unal.edu.co; 4 Hospital Infantil de México Federico Gómez, Dr. Márquez 162, Col. Doctores, Cuauhtémoc, Ciudad de México C.P. 06720, Mexico; luzmrr7@yahoo.com.mx; 5 Departamento de Ingeniería Química, Facultad de Ciencias Químicas, Universidad Autónoma de Coahuila, Blvd. V. Carranza e Ing. José Cárdenas V. S/N, Saltillo, Coahuila C.P. 25280, Mexico; a_romero@uadec.edu.mx

**Keywords:** diacetylenes, push-pull polymers, non-linear optics, optical materials, organic films

## Abstract

Excellent quadratic non-linear optical (ONL-2) properties of the poly(2,5-*bis*(but-2-ynyloxy) benzoate, containing a polar diacetylene as a chromophore, were found. According with the Maker fringes method, oriented polymer films showing an order parameter of ∼0.23 can display outstanding and stable Second Harmonic Generation (SHG) effects under off-resonant conditions (SHG-532 nm). Also, the macroscopic non-linear optical (NLO)-coefficients were evaluated under the rod-like molecular approximation, obtaining: *χ_zzz_*^(2)^ and *χ_zxx_*^(2)^ in the order of 280 ± 10 and 100 ± 10 pm V^−1^, respectively. The mechanical and chemical properties, in addition to the large ONL-2 coefficients exhibited by this polymer, make it a promising organic material in the development of optoelectronic/photonic devices.

## 1. Introduction

Polydiacetylenes (PDAs) are a unique class of linear conjugated polymers derived from the topochemical polymerization of diacetylene (DA) monomers. One important aspect of their behavior is that they can aggregate into large-dimensional crystals. [1,2,3]. Wegner’s pioneering work demonstrated that DA monomers undergo topochemical polymerization to form PDAs [4]. 

These compounds are synthesized via a 1,4–addition reaction of monomer crystals of the form R–C≡C–C≡C–R, where the polymer backbone consists of alternating their alkene–alkyne structures. Owing to their intriguing stress-induced chromic transition (blue to red) and non-linear optical properties (NLO), PDAs have been extensively investigated as potential chemosensors and photonic materials. Recently, the embedment of PDA and its role in guest-host supramolecular chemistry has been used advantageously for the assembly of well-defined structures with unique optical, electronic, and mechanical properties [5]. NLO in organic polymers has been considered as a very promising research field, with potential applications in opto-electronics and photonics materials; this one is due to the possibility of designing multifunctional materials at the molecular level with specific properties and at lower costs. Such properties may include strong and ultrafast non-linear optical responses, better processability characteristics and high potential for its incorporation into photonic/optoelectronic devices [6,7,8,9]. 

Considering quadratic NLO-applications such as SHG within the dipolar approximation, rod-like organic compounds display NLO-activity if they include conjugated molecules within their molecular architectures. Such conjugated moieties must contain substituents that bestow an important degree of push-pull effect unto them via electron donating (D) and electron accepting (A) functional groups. Besides this, in the case of polymers, these conjugated portions should be contained peripherally. This molecular design is commonly implemented for obtaining functionalized polymers suitable for second-order SHG/NLO-applications. Here, due to the push-pull effect induced upon photoexcitation (via the driving force of the laser electric field and the donor-acceptor functional groups), a modification in the charge distribution is forced; thus, this results in an intramolecular charge transfer and the subsequent NLO-response [10,11]. 

Many efforts have been devoted to the development of second-order NLO materials; particularly to the efficient translation of large molecular first hyperpolarizability to high *d*_33_ SHG macroscopic coefficients. The main obstacle in this area has been the chromophore aggregation in the solid state (film samples), since the NLO chromophores are often highly polarized via the DA push-pull interactions. This effect causes the phenomenon that during the film formation, chromophores with large dipole moments tend to compactly pack, leading to the diminishment or cancellation of the dipoles and their assembling in centrosymmetric arrangements, which decreases their NLO properties. Besides, steric factors, polarization of the polymerizable groups by substituents and the ability of the substituents to participate in resonance stabilization, influence the reactivity. Additionally, a high glass transition temperature leads to a lower degree of conversion [6,8,11,12]. Nevertheless, a strict requirement for inducing quadratic NLO-effects in organic materials relies on the arrangement being non-centrosymmetric. On a molecular level, being non-centrosymmetric is quite easy to achieve by connecting strong electron donors and acceptors by a conjugated π-bridge, yielding strong dipolar molecules [10]. 

Moreover, the incorporation of the NLO-active chromophores to a polymer backbone by covalent bonding (side chain polymers) or into a polymer main chain generally results in an enhanced chromophore density, without phase separation, within the whole polymeric structure, and therefore, in higher NLO effects [13]. Following this approach, polymer films with NLO chromophores doped or synthetically attached to the polymer backbone represent the main subject of recent studies, for instance, gaining insight about the optical limiting performances of substituted polydiacetylenes with a varied spectrum of functional groups [8,11,14,15,16]. 

Conjugated polymers are generally characterized by highly anisotropic optical, dielectric, conducting and mechanical properties. The reason for this is that valence electrons respond more easily to perturbations along directions where the conjugation occurs, whereas the delocalization in other directions is hindered by saturated bonds. This one induces an isolator type behavior across the conjugation direction, but a semiconducting behavior along that direction. In this way, PDAs can be modeled as one-dimensional, organic semiconductors, and readily obtained in form of large, nearly defect-free single crystals [1]. 

The introduction of aromatic portions containing groups providing a push-pull effect is a regular strategy to improve the NLO-behavior of PDAs, due to the increase and extension of electronic conjugation [2,11,17]. Unfortunately, the monomeric DAs are often non-reactive, or the corresponding polymer is not soluble in common organic solvents, leading to material instability and/or unprocessability, which have significantly limited their use in practical technological applications [2,18,19,20]. Given these problems, and in order to explore practical alternatives to the synthesis of PDAs-based structures [17,21], we synthesized a highly soluble conjugated 2,5-bis(but-2-ynyloxy)benzoate polymer, which contains covalently bonded chromophores as side chains (nitro (pull) and amino (push) functional groups), and its photophysical properties, including the development of PDAs networks, thin films and their quadratic SHG/NLO-characterization.

## 2. Results and Discussion

### 2.1. Synthesis of Polymer and Total Material Characterization

**Scheme 1:**
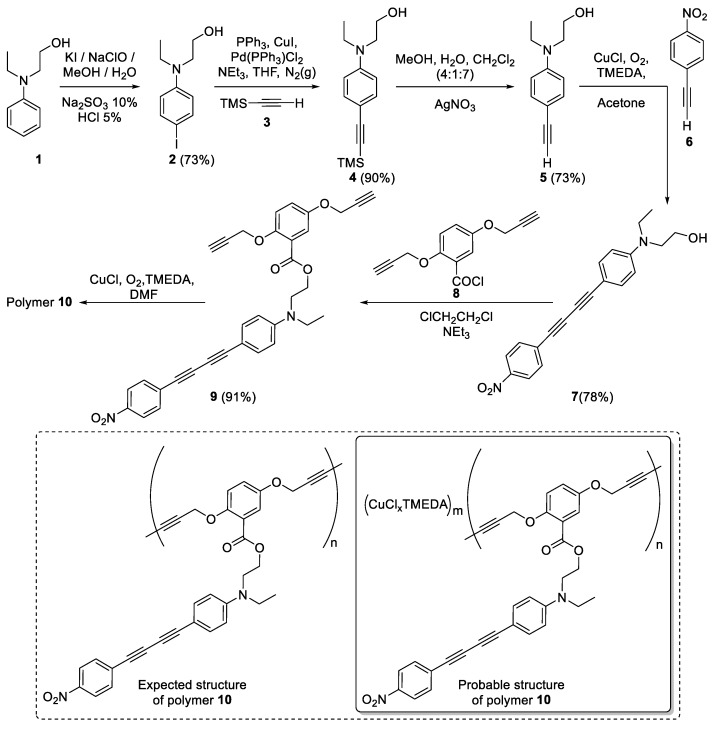
Synthetic route towards the polymer **10**.

The synthesis of polymer **10** from 2-(ethyl(phenyl)amino)ethanol (**1**) was previously published, [21]. Now we show our results of the synthesis of polymer **10** which we show in Scheme 1, the yield for compound **2** was improved from 57% to 73%. This was achieved through a better iodination technique. Another enhancement was achieved for compound **5**, from 67% to 73%, thanks to the manipulation of the reaction media. The total yield and shortening of the total synthetic route was achieved by acquiring compound **6** instead of synthesizing it. Chromophore **7** was also obtained in a better yield from 70% to 78%.

**Figure 1:**
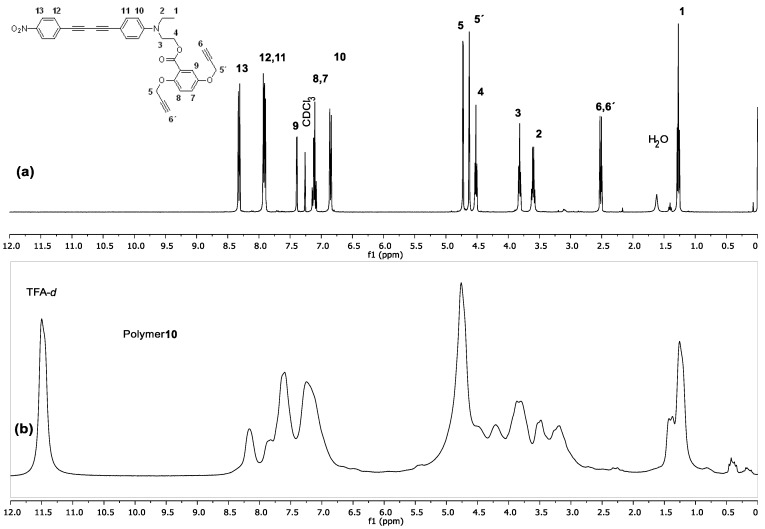
(**a**) ^1^H nuclear magnetic resonance (NMR) spectrum of **9** in deuterated chloroform (CDCl_3_) and (**b**) ^1^H NMR spectrum of **10** in TFA-*d*.

**Figure 2:**
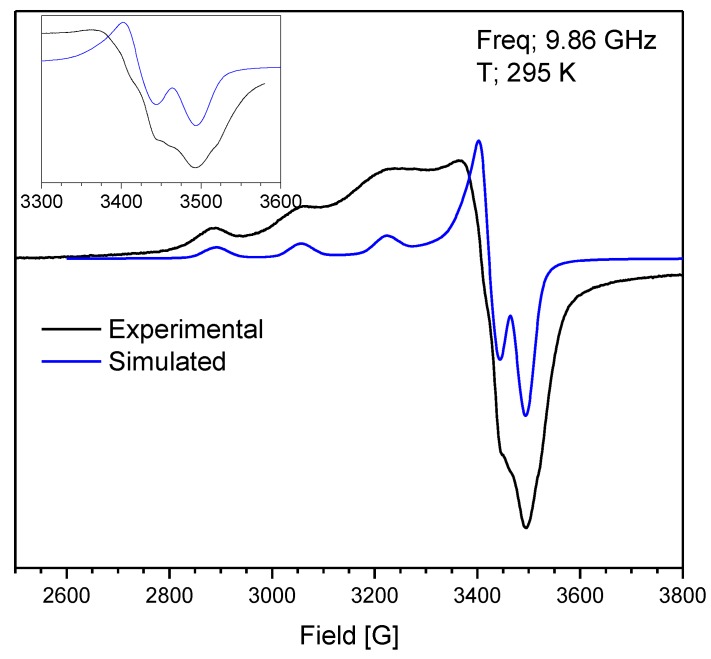
Experimental and simulated electron paramagnetic resonance (EPR) spectra at room temperature (295 K) of a powder sample of polymer **10**. Medium wave (MW) Frequency: 9.865 GHz; Mod. Modulation: 100 KHz.

In general, the synthetic route was improved in its total yield and effectiveness in obtaining the chromophore **7**, which is the key part of the synthetic design. The ^1^H nuclear magnetic resonance (NMR) spectrum of the polymer **10** has not been previously reported, but a comparison between the ^1^H NMR spectrum of the **9** (Figure 1a) and the **10** (Figure 1b) was done. The ethynyl protons at 2.50 (6) and 2.53 ppm (6′) of **9** disappear just after the oxidative coupling polymerization. The latter fact indicates that no residual ethynyl protons due to unreacted acetylene groups are present in the obtained polymer **10**. The signals between 8.16–7.24 ppm correspond to the aromatic groups, 4.76–3.78 are likely to correspond to the aliphatic groups, and 1.26 ppm corresponds to a methyl group of **10**. Nevertheless, the signals between 3.54–3.18 ppm could be a fraction of Tetramethyl-ethylenediamine (TMEDA) incorporating to the macrostructure. In order to clarify the structure of the polymer, we carry out the electron paramagnetic resonance (EPR) spectroscopy of polymer **10.** The Figure 2 shows the experimental (black line) and simulated (blue line) EPR spectra at room temperature (295 K) of **10**; we can observe Cu(II) species in the sample of **10**. The EPR spectrum shows an axial spectrum characteristic of Cu(II) species with g-values of 2.245 and hyperfine constant of 1.7 × 10^−2^ cm^−1^ assigned to the Cu(II) center in the parallel component, while for the perpendicular component, a g-value of 2.056 and hyperfine constant of 1.4 × 10^−3^ cm^−1^, which best-fit to 2 Nitrogen centers. A g-average of 2.119 was obtained. The EPR spectrum of **10** is similar to the EPR spectra for CuCl_2_TMEDA reported by Bai et al. [22]. Therefore, we conclude that the polymer **10** contains Cu(II)Cl_x_TMEDA (where x = 0, 1, 2), but at this time we are doing experiments to be able to elucidate the macrostructure of polymer **10**.

A GPC-analysis with polymetylmetacrylate standards shows that this polymer has a molecular weight of 12,560 (*Mn*) and a polydispersity index of 1.5. The molecular weight of this polymer has not increased, although the polymerization reaction was maintained for several days. When increasing the chain length, starting from just a few repeat units, it is an important question, at which molecular weight the (macro) molecule develops the typical characteristics of a polymer [23]. The most significant difference between polymers and other materials appears in the viscoelastic properties at times longer than the structural relaxation time [24]. 

**Figure 3:**
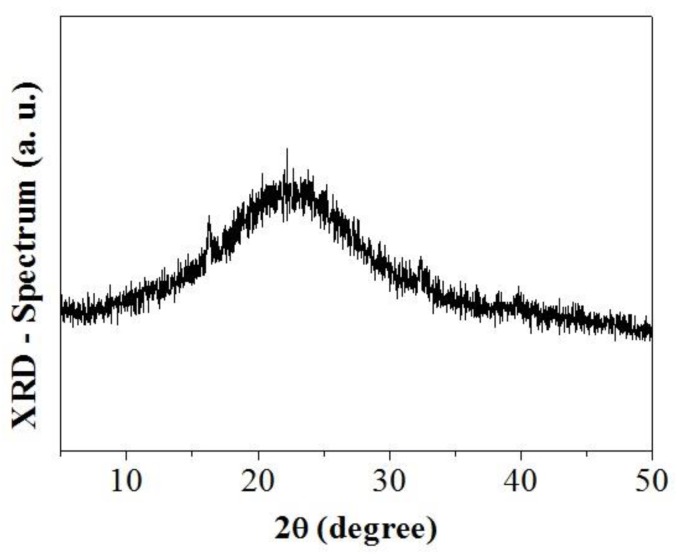
X-ray diffraction (XRD) pattern of **10**.

Thus, by changing the molecular weight of a chain *Mn*, there can be observed a transition from the typical behavior of small molecules to the characteristic Rouse dynamics of polymers. This transition should occur at a specific *Mw* value, above which molecules exhibit Gaussian statistics and then become “polymers” [24,25,26,27]. The aromatic diacetylenes are designed to be more soluble and mechanically flexible and less thermally reactive [28]. In fact, according to several authors [29], polymers comprising diacetylene groups in their main molecular chain and obtained via the Hay synthetic route, afford polymers with relatively low molecular weights, this being in agreement to what we have obtained previously; this is because this effect can be explained through cyclization reactions between terminal alkyne portions. Indeed, such behavior may be due to the sterically-hindered chromophore groups, which keep the polymer from growing. The polymer is amorphous in nature, as judged from its powder X-ray diffraction pattern (Figure 3); usually an amorphous polymer exhibits good solubility and allows the fabrication of transparent films.

**Figure 4:**
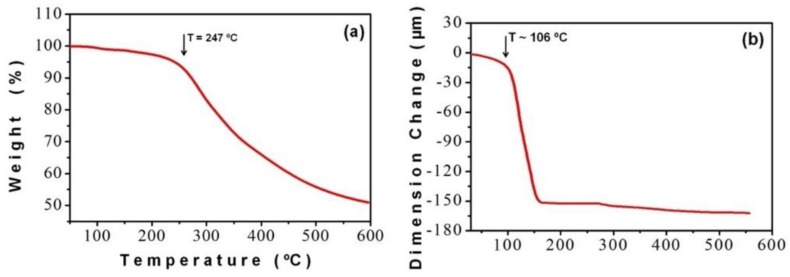
(**a**) Polymer thermal properties: Thermogravimetric analysis (TGA)-diagram and (**b**) thermomechanical analysis (TMA)-diagram.

Thermal stabilities by thermogravimetric analysis (TGA) measurements show that the decomposition temperature (5% weight loss) of the polymer **10** was found at ~247 °C. Thus, the onset of the main exotherm (~149 °C) occurred at temperatures before any significant degradation or weight loss process (Figure 4a). Indeed, the polymer exhibits a good thermal stability as required for thin films preparation procedures and optical inspections demanding energetic pulsed laser irradiation. From the thermomechanical analysis (TMA)-diagram (Figure 4b) it can be seen that the value of *T*_g_ for polymer **10** is 106 °C. As it has been seen before, corona-poling experiments must be carried out above the *T*_g_ value of each particular polymer [30]. The polymer was obtained as orange fibers showing high solubility in typical organic solvents (such as NMP, THF, DMSO and DMF).

### 2.2. Morphological and Spectroscopic Film Characterizations

**Figure 5:**
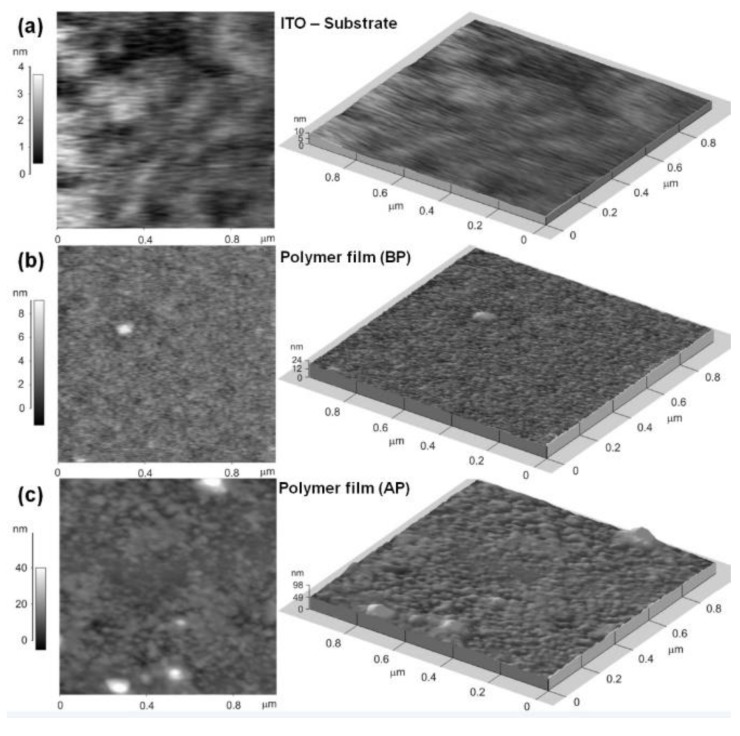
2D- and 3D-atomic force microscopy (AFM)-scanned micrographs (same length-scale): (**a**) A naked indium tin oxide (ITO)-coated glass substrate, (**b**) an unpoled (BP) polymeric film deposited onto an ITO-substrate and (**c**) a poled polymeric film sample (AP).

Through a typical spin-coating methodology, optical quality films suitable for spectroscopic characterizations were produced. Figure 5 shows 2D and 3D atomic force microscopy (AFM)-micrographs, depicting the packing structure, film topography and surface morphology of poled and unpoled films. 

Figure 5a shows the surface morphology of a bare indium tin oxide (ITO)-coated glass substrate depicting consistent strip-like morphology at the nanometric scale, with no important irregularities. The obtained root-mean-square (RMS) roughness is on the order of 5.1 Å, and is mainly produced by elongated nanometric arrangements. On the other hand, the spin-coated polymer/NMP films deposited onto the ITO-glass substrates exhibit different topographies (Figure 5b). Granular material arrangements appear on the surface morphology, indicating an unvarying polymer covering on the ITO-film and to a rougher surface (rms roughness: ~7.06 Å). In this case, a stable and relatively homogeneous texture with orderly grain-size distribution at the nanometric scale can be detected. This is evidence of close-packed structures and long-range order. 

Finally, the surface texture of the electrically poled polymeric films exhibits drastic variations on its morphology; such changes are basically triggered by the induced molecular reordering via intense DC-fields (Figure 5c). In this case, the uniformity of the surface morphology is reduced, and several irregularities and/or prominent enlargements of the granular structures tend to appear as measured from high resolution digitalized images; at least a bimodal grain size distribution is observed, the smallest one (irregular grains in the order of ~20–40 nm) being the most prominent. This lack of uniformity and long-range order gives rise to larger and irregular flattened structures, leading to a significant increase of the rms roughness: ~39.3 Å. According to the literature [10,31], these features exemplify the structural film evolution triggered by the electrical poling performed at high temperatures and permanent thermal processes. Because of this, the polar molecules or functional groups may be displaced within the film layer, leading to a surface reordering as the interacting dipoles tend to orient in opposite directions, but the electric field forces them to align along its direction. Moreover, after electrical poling is carried out, crystal growth within the host matrix and shifts of the polymeric network resulting from cross-linking (activated by heating) can also alter the film morphology. Generally, all these effects establish a clear influence on the structural properties of the organic films and their corresponding opto-electronic properties.

**Figure 6:**
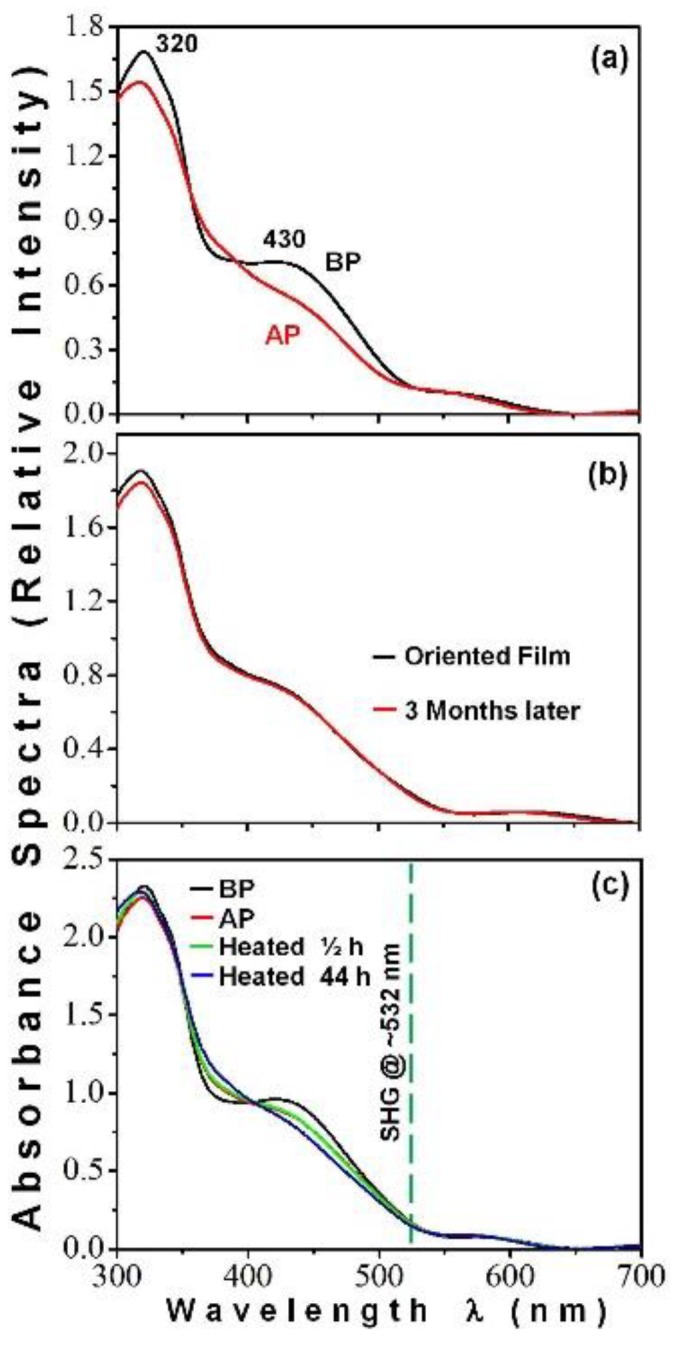
Ultra Violet (UV)-Visible absorbance spectra of polymeric films: (**a**) Recorded before and after performing electrical poling (at 120 °C for 5 h), (**b**) Variations on the absorbance spectra of a poled film after several months (sample was stored at room conditions) and (**c**) Variations on the absorbance spectra of a poled film after applying a thermally-induced molecular relaxation process (at 80 °C for 30 min and 44 h).

The molecular orientational order of poled films can be evaluated from the UV-Vis absorbance spectroscopic measurements. In fact, the UV-Vis spectral features of unpoled polymeric samples (Figure 6a) exhibit two strong absorption bands, namely at 320 and 430 nm, arising from the aromatic groups with π–π* transitions and from nitro group with n-π* transitions, respectively. However, after performing electrical poling, an intensity decrease in these bands is recognized. Regarding the drastic decrease of the band at 430 nm after performing electrical poling, it can be mechanically understood according to the molecular reorientation induced by the external electric field; in fact, before the poling procedure, the molecules are randomly oriented on the substrate’s surface, and the absorption properties of the polymeric net are considerably high, whereas in the electrically poled film the molecules are parallelly reorganized in the direction of the external field (close to perpendicular to the substrate’s surface), so that that the pendant groups exhibit a pi-stacking and the interaction with the electric field of the incident light is lower, thus the corresponding absorption properties/molar absorptivity of these poled films drastically decreases, causing the observed hypochromic effect. On the other hand and in terms of electronic transitions, it is observed that the band at 320 nm (corresponding to aromatic groups with π–π* transitions) slightly decreases after performing electrical poling; this is due to the high probability associated to these relatively energetic transitions in organic compounds, which is practically not affected by the induced molecular reorientation, whereas the band at 430 nm (corresponding to the less energetic n-π* transitions) practically disappears in the polymeric poled film due to its lower probability. In fact, these transitions are drastically affected by the molecular reorganization induced by the electrical poling and the decrease of the molecular absorption cross-section in a quasi-parallel molecular configuration. 

The order parameter or molecular alignment *φ* can then be determined from the following relation: *φ* = 1 − (A_2_/A_1_), 0 < *φ* < 1; where A_1_ and A_2_ denote the absorbance intensities of the main bands measured before and after performing electrical poling, respectively. Accordingly, organic films made from **10** were able to support outstanding stable molecular ordering with an average order parameter of *φ* ≈ 0.23. Indeed, after several months, the poled films still exhibit highly-ordered molecular structures, excellent transparency properties and high solubility, allowing consistent photophysical studies (Figure 6b). There are variations in the molecular orientation between these poled films since we have implemented several samples in our studies (for different purposes); thus variations in the absorption values arise from the different order parameter and thicknesses of the samples. On the other hand, the main evidence of the decrease of the absorption band at 430 nm is the fact that this flattened and distorted spectral band drastically changes its shape (after poling) to a less pronounced band with a monotonically decreasing behavior (in both cases: Figure 6a,b) and without the flattened structure to the UV range. Moreover, the thermal relaxation performance of the poled films was also studied by heating the samples up to 80 °C for ~44 h, and the variations of the absorbance spectra in time were monitored (Figure 6c). Results show a slow decrease in the molecular orientation for the poled films. Nonetheless, after 30 min of heating, no further decrease was observed, and the order parameter attained a terminal value *φ* > 0. This effect is probably due to chemical modifications or to mechanical structural modifications arising throughout the poling process at high temperatures; such effects may also produce moderate molecular cross-linking reactions, so that several chromophores remain permanently orientated. Certainly, if a polymer main-chain is easily distorted throughout the poling process due to a flexible molecular architecture, the effective alignment efficiency of the polymer/chromophore system is low. Consequently, the chromophore relaxation will instantaneously take place, leading to a terminal order parameter of *φ* ≈ 0. Conversely, in the poled films, chromophores attached to the polymer main-chain remain highly ordered for a long time, demonstrating an outstanding rigid polymeric main-chain structuring. This configuration cannot be easily deformed after a final aligned arrangement has been reached (even under heating conditions beyond the *T_g_*-value). Finally, providing that polymeric films remain fully soluble in DMF and NMP, no cross-linking effects are associated with the permanent chromophore alignment. 

Hence it is concluded that the mechanical stability of this polymer can be explained in terms of its inherent main-chain rigidity. Indeed, according to Huang and coworkers [32], the introduction of a biphenylene group may efficiently decrease intermolecular electrostatic interactions, stimulating the chromophore alignment.

### 2.3. Quadratic SHG/NLO-Photophysical Characterizations

The NLO/SHG-properties (*χ*^(2)^-response) of the polymeric film samples were investigated, implementing the following physical model which has been described previously [29]:

(1) $$I_{2\omega }\left(\theta \right)\approx \frac{512\pi^{5}}{c\lambda^{2}n_{2\omega }^{2}}\frac{t_{1}^{4}t_{2f}^{2}t_{2s}^{2}}{\cos^{2}\theta_{2\omega }}\frac{I_{\omega }^{2}}{A}{\left(L^{Film}\chi_{eff}^{\left(2\right)}\right)}^{2}\frac{\sin^{2}\Phi }{\Phi^{2}}$$

(2) $$\Phi \equiv \frac{2\pi L^{Film}}{\lambda_{\omega }}\left(n_{\omega }\cos\theta_{\omega }-n_{2\omega }\cos\theta_{2\omega }\right)$$

(3) $$\chi_{eff_{S\text{-}In/P\text{-}Out}}^{\left(2\right)}≅\chi_{zxx}^{\left(2\right)}\sin\theta_{2\omega }$$

(4) $$\chi_{eff_{P\text{-}In/P\text{-}Out}}^{\left(2\right)}≅\chi_{zxx}^{\left(2\right)}\left(\cos\theta_{2\omega }\sin2\theta_{\omega }+\sin\theta_{2\omega }\cos^{2}\theta_{\omega }\right)+\chi_{zzz}^{\left(2\right)}\sin\theta_{2\omega }\sin^{2}\theta_{\omega }$$

where: *I_ω_* and *A* are the intensity and spot-area of the focused fundamental beam, respectively; *t*_1_, *t*_2*f*_ and *t*_2*s*_ represent the fundamental and SHG Fresnel transmission coefficients (Equation (1)); the phase angle *Φ* can be expressed as (Equation (2)) [29,33,34,35,36,37,38] *n_ω_* (*n*_2*ω*_), representing the film refractive index at the fundamental (second harmonic) frequency; *θ_ω_* (*θ*_2*ω*_) is the refractive angle of the fundamental (second harmonic) wave determined by sin*θ* = *n_ω_* sin*θ_ω_* = *n*_2*ω*_ sin*θ*_2*ω*_. In our system, the rod-like oriented chromophores allow the determination of the *χ_zzz_*^(2)^ and *χ_zxx_*^(2)^ independent NLO-coefficients. Accordingly, given that the SHG-waves were P-polarized regardless of the polarization state of the fundamental waves, only two dependent equations were obtained for the NLO-coefficients: (a) For the S-polarized fundamental beam (Equation (3)) [36,37,38]; and (Equation (4)) for the P-polarized fundamental beam.

**Figure 7:**
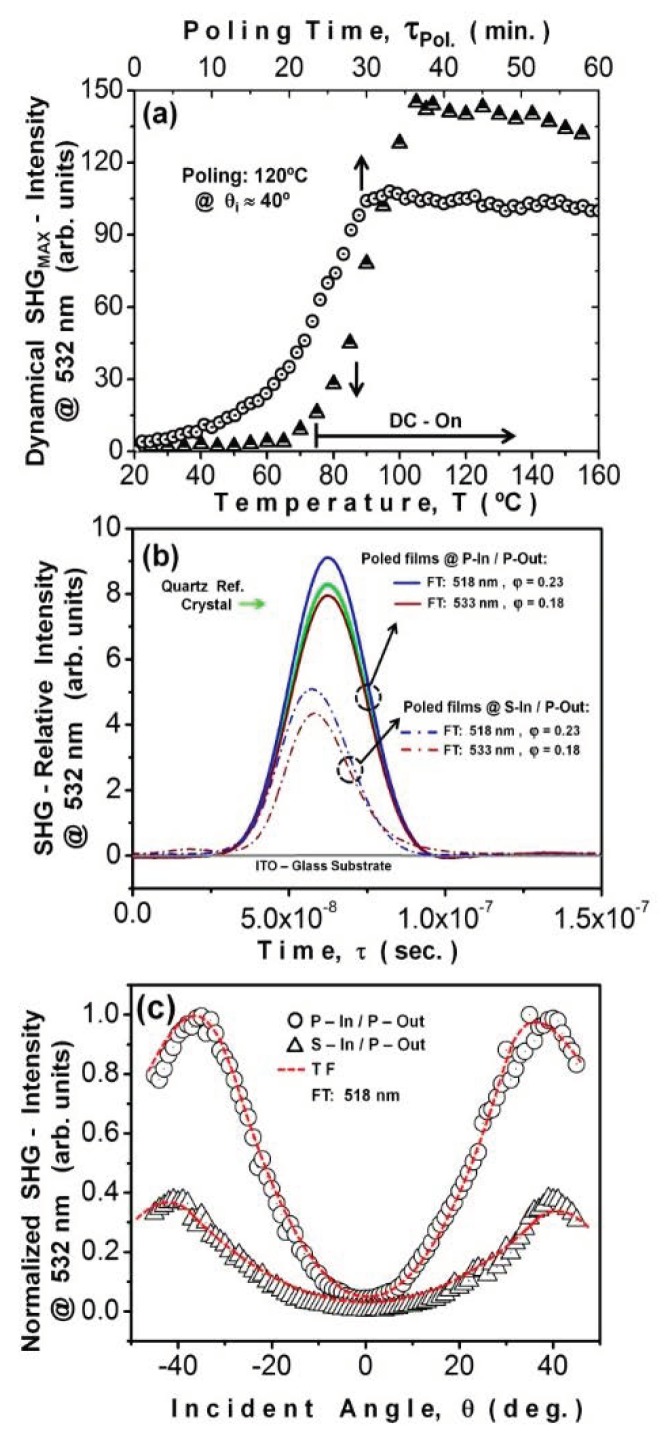
(**a**) In-situ Second Harmonic Generation (SHG)-intensity signals recorded at an incident angle of ∼40° (@SHG_MAX,_ is the maximum value of the SHG signal al best phase matching conditions) for a polymeric poled film; (**b**) Averaged SHG-intensity signals and (**c**) Angle-dependent SHG-signals (Maker fringes).

In the Figure 7a, is possible observe the increase of the temperature of the sample **10**, but no SHG-signals were detected within the 25–75 °C (DC-field: OFF). However, as the corona poling procedure is started (75–160 °C, DC-field: ON), measurable SHG-signals with highest intensities at ~100–110 °C were detected. Furthermore, according to dynamical measurements, the SHG-signals remain practically unchanged after ~30 min of electrical poling (optimal poling temperature: 120 °C). Based on these poling parameters, various film samples were optimally poled and cooled-down to perform NLO/SHG-characterizations at room temperature. 

According to Figure 7b, experiments were carried out for two selected poled films (thicknesses: 533 and 518 nm) and their characteristic SHG averaged signals (obtained from both P-In/P-Out and S-In/P-Out polarizing geometries) were directly recorded at “best possible” optical phase-matching; for example, at maximal SHG output intensities (incidence angle: ~40°).

The Figure 6a–c show that the absorbance spectra of both poled and unpoled films, at the fundamental and SHG frequencies (*λ_ω_* = 1064 nm/*λ*_2*ω*_ = 532 nm), are reasonably far from resonant absorbance conditions (*λ_max_* < 320 nm), whereby Equations (1)–(4) may reasonably be used for the evaluation of the SHG-signals and the macroscopic *χ*^(2)^-coefficients.

Accordingly, the SHG coherent emissions unequivocally confirm an average non-centrosymmetric macroscopic structuring of the poled polymeric films. Besides, the Figure 7c shows the distinctive angle-dependent SHG-signals (Maker fringes) measured from the best poled film (film thickness: FT ≈ 518 nm, *φ* ≈ 0.23) in P-In/P-Out and S-In/P-Out polarizing geometries [29,33]. 

The SHG data observed from the P-In/P-Out geometry was stronger than that observed for the S-In/P-Out configuration, with a [P-In/P-Out]/[S-In/P-Out] signal ratio of ~3.3. This fact evidences the prominence of the *χ*^(2)^_33_ NLO-coefficient over the *χ*^(2)^_31_ one, which testifies to the NLO interactions of the long and short molecular axes of the constituting rod-like chromophores [9,29,33], as well as to the current chromophore alignment.

Finally, in order to estimate the tensorial coefficients of the NLO-chromophore, the SHG-signal calibration was performed according to the relative Maker fringes method. Here, an α-quartz crystal was used as NLO-reference material. In this way, by modeling the film sample as a four-layer structure (Air-Film-Substrate-Air), and considering the optical absorbance negligible at both fundamental and SHG-frequencies, non-resonant experimental conditions are assumed (Figure 7c). 

Thus, classical, theoretical models can be implemented assuming the Kleinman symmetry relations [34,39,40]. Hence, the SHG-intensity *I*_2*ω*_(*θ*) as function of the incident angle *θ_i_* may obey the expression (**1**) [36,37,38].

After performing the calibration procedure of the experimental device by implementing the reference *α*-quartz plate, the signal ratio was fixed for both the reference crystal and film samples. Thus, the evaluation of the tensorial NLO-coefficients was performed by fitting the respective theoretical expressions to the experimental data (Figure 7c).

After simultaneous fitting, we obtained: *χ_33_*^(2)^ = *χ_zzz_*^(2)^ ≈ 280 ± 10 pm V^−1^ and *χ_31_*^(2)^ = *χ_zxx_*^(2)^ ≈ 100 ± 10 pm V^−1^. The *χ_ij_*^(2)^ values are relative high considering the film thickness, and larger than those reported for typical poly(acetylene), poly(diacetylene), poly(styrene), polybenzoates and poly(methylmethacrylate)-based polymers containing azo-compounds and tolan-units, as NLO-chromophores [9,29,33,41,42]. These results point out promising/potential applications for this kind of polymer containing polar diacetylenes. In fact, to the best of our knowledge, no scientific reports on the quadratic NLO/SHG-properties of **10**, containing polar diacetylene-units as pendant NLO-active chromophores, have been reported. Finally, as shown in the Figure 7a, the poled organic films exhibit important NLO/SHG thermal and temporal stability properties. As a matter of fact, our poled films preserve their SHG-conversion efficiency (above 90%) for long periods of time (including several years). These poled films even show strong SHG-intensities for temperatures well above the *T_g_* value; for instance, the materials herein described were able to exhibit SHG-conversion efficiencies of ~80% at temperatures close to 160 °C, demonstrating the rigidity, strength and thermal stability of the polymeric main chain.

**Figure 8:**
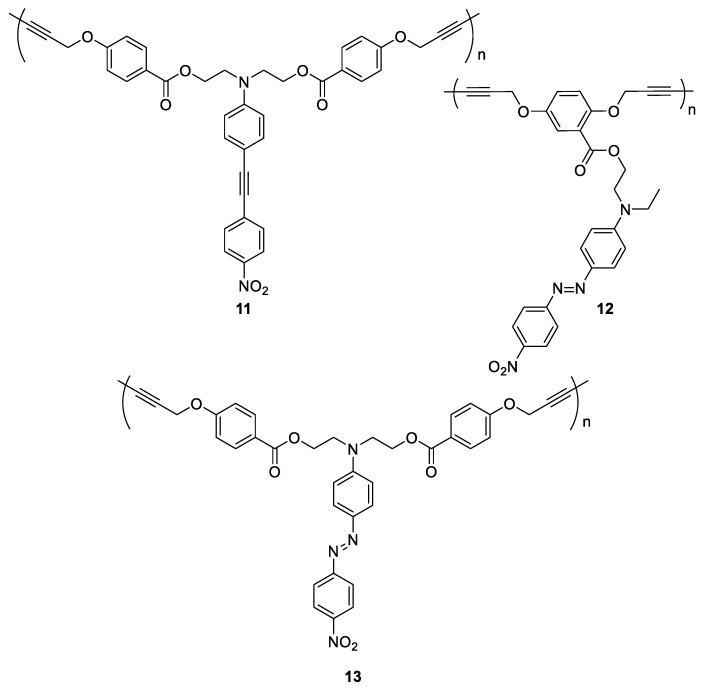
Polymer structures containing NLO-coefficients the second order.

Analyzing NLO-coefficients, the second order of polymer **10** and other polymers reported (Figure 8) are showed on the Table 1; by comparing **11** and **13** we can say that, considering they posses the same main chains, the change relies on the chromophore portion, and comparing between the diacetylene and diazo entities, the former is the one presenting the highest NLO responsiveness [43]. In the same manner, if we compare **12** and **13** we have the same diazo chromophore, and the part that changes is the main chain, and seeing the values of NLO responsiveness, we can observe that the chromophore is the chemical entity that bestows the NLO properties. In addition, from our experience, the diacetylene pi-bridge provides more conjugation to the chromophore, and thus, better NLO responsiveness can be achieved as we present using polymer **10**. Based on what is mentioned above regarding the conjugation of the pi-bridge with the diacetylene moiety, responsible for high values of second order NLO properties, we decided to use that as a criterion to choose a chromophore with that kind of conjugation, such as is our polymer **10**, and comparing it with polymer **11**, one that has only one acetylene. However, the high NLO values of polymer **10** cannot only be due to additional acetylene within the chromophore pendant group. Undoubtedly, the existence of Cu(II)Cl_x_TMEDA in polymer **10** is affecting the NLO values. Currently, we are doing experiments to know what is the role of copper in the polymer **10** in NLO values.

## 3. Experimental Section

### 3.1. Materials and Instrumentation

All products were characterized by spectroscopic techniques. MS was performed implementing an Orbitrap Fusion TM Tribrid spectrometer and Jeol JMS-5 X 102 A instrument. IR-spectra were acquired in a FT-IR/ATR Nicolet 6700 spectrometer using a SeZn glass in contact with the sample surface. ^1^H NMR and ^13^C NMR-spectra were recorded on a Bruker Advance 400-MHz spectrometer using TMS as an internal standard. UV-Vis spectroscopy was performed in a Varian Cary 400 Cone UV-Vis spectrometer. The molecular weight of the obtained polymer was determined by gel permeation chromatography (GPC) relative to polymetylmetacrylate standards in DMF at 25 °C on a Waters 2695 ALLIANCE separation module. TGA-analysis was carried out with an SDT-Q600 apparatus (TA-Instruments, 25–600 °C). To determine the melting point of the processed materials, a Fisher-Johns apparatus was used. These latter measurements were performed with a heating rate of 25 °C min^−1^.

### 3.2. Synthesis and Characterization of the Monomer and then, the Polymer

The detailed description of the methods of the preparation of analog compounds has been previously described [29], and we only reported spectroscopic data (except reagent **6**, which was purchased directly from Aldrich). In addition, we describe how we performed the deprotection of **4**, which was carried out with a solution of AgNO_3_.

#### 3.2.1. 2-(ethyl(4-iodophenyl)amino)ethanol (**2**)

The solid was purified by crystallization (hexane) to provide the desired product in 73% yield (from **1**), as white crystals. ^1^H NMR (400 MHz, CDCl_3_) *δ* (ppm): 1.13 (t, 3H, *J* = 7.2 Hz, CH), 1.77 (s, 1H, OH), 3.39 (q, 2H, N-CH_2_), 3.41 (t, 2H, *J* = 6.0 Hz, N-CH_2_), 3.75 (t, 2H, *J* = 5.4 Hz, CH_2_-OH), 6.51 (d, 2H, *J* = 7.2 Hz, CH-Ar-N), 7.43 (d, 2H, *J* = 7.2 Hz, CH-Ar-OH). ^13^C NMR (100 MHz, CDCl_3_) *δ* (ppm): 11.9 (CH_3_), 45.8 (CH_2_-N), 52.6 (CH_2_), 60.3 (CH_2_-OH), 77.4 (OH), 114.9 (CH-Ar-N), 137.9 (CH-Ar-I), 148 (C-N). FT-IR, *υ_max_* (cm^−1^): 3103, 1587, 804 (Ar-H, Ar-C), 3323, 1047 (O-H, C-OH), 2968, 1587 (CH_3_), 2933, 1496 (CH_2_), 1356 (Ar-NO_2_), 503 (C-I). mp = 47–48 °C.

#### 3.2.2. 2-(ethyl(4-((trimethylsilyl)ethynyl)phenyl)amino)ethanol (**4**)

The compound was purified by a wash of hot hexane to obtain a light yellow liquid with a yield: 90% (from **2**) ^1^H NMR (400 MHz, CDCl_3_) *δ* (ppm): 0.20 (s, 9H, Si-CH_3_), 1.15 (t, 3H, *J* = 7.0 Hz, CH_3_), 1.85 (s, 1H, OH), 3.42 (c, 2H, *J* = 7.1 Hz, N-CH_2_), 3.47 (t, 2H, *J* = 6.0 Hz, N-CH_2_), 3.78 (t, 2H, *J* = 6.0 Hz, CH_2_-OH), 6.62 (d, 2H, *J* = 8.8 Hz, CH-Ar-N), 7.32 (d, 2H, *J* = 9.2 Hz, CH-Ar-C≡C). ^13^C NMR (100 MHz, CDCl_3_) *δ* (ppm): 0.19 (Si-CH_3_), 12.1 (CH_3_), 45.7 (N-CH_2_), 52.5 (CH_2_), 60.4 (CH_2_-OH), 91.5 (C≡C-Si), 106.6 (Ar-C≡C), 110.2 (C-Ar), 119.9 (CH-Ar-N), 133.5 (CH-Ar-C≡C), 148.3 (C-N). FT-IR, *υ_max_* (cm^−^^1^): 3043, 1606, 818 (Ar-H, Ar-C), 3381, 1047 (O-H, C-OH), 2962, 1516 (CH_3_), 2895, 1456 (CH_2_), 2148, 1398 (C≡C), 1249, 843 (C-Si), 1356 (C-NO_2_).

#### 3.2.3. 2-(ethyl(4-ethynylphenyl)amino)ethanol (**5**)

Compound **4** (2 g, 7.65 × 10^−3^ mol) was dissolved in a homogeneous reaction mixture using dichloromethane (24 mL), methanol (14 mL) and water (4 mL) in the following relation 7:4:1, respectively. Thereafter, silver nitrate (AgNO_3_, 2 g, 0.1 equiv.) was added to the mixture reaction. In this mixture, the deprotection reaction was achieved at 40 °C for 24 h. Finally, an aqueous saturated solution of ammonium chloride (NH_4_Cl) was added. A brown mixture was obtained and extracted with dichloromethane (150 mL × 6). The obtained organic phase was dried with MgSO_4_, concentrated and vacuum dried. The product was purified by crystallization (95:5 hexanes/CH_2_Cl_2_) to provide the desired product in 73% yield (from **4**) as light yellow needle crystals. ^1^H NMR (400 MHz, CDCl_3_) *δ* (ppm): 1.17 (t, 3H, *J* = 8.6 Hz, CH_3_), 1.69 (s, 1H, OH), 2.98 (s, 1H, C≡H), 3.43 (q, 2H, *J* = 8.7 Hz, CH_2_), 3.48 (t, 2H, *J* = 5.8 Hz, N-CH_2_), 3.80 (t, 2H, *J* = 5.6 Hz, CH_2_-OH), 6.66 (d, 2H, *J* = 8.8 Hz, CH-Ar-N), 7.35 (d, 2H, *J* = 8.8 Hz, CH-Ar-C≡H). ^13^C NMR (100 MHz, CDCl_3_) *δ* (ppm): 11.9 (CH_3_), 45.5 (N-CH_2_), 52.3 (CH_2_), 60.2 (CH_2_-OH), 74.8 (Ar-C≡C), 84.7 (C≡C-H), 108.9 (Ar-C), 111.8 (CH-Ar-N), 133.5 (CH-Ar-C≡H), 148.3 (C-N). FT-IR, *υ_max_* (cm^−1^): 3049, 1595, 815 (Ar-H, Ar-C), 3378, 1046 (O-H, C-OH), 2967, 1516 (CH_3_), 2927, 1399 (CH_2_), 3289, 2095 (C≡H), 1355 (Ar-NO_2_). mp = 45–47 °C.

#### 3.2.4. 2-(ethyl(4-((4-nitrophenyl)buta-1,3-diynyl)phenyl)amino)ethanol (**7**)

The product was purified by crystallization (1:1 hexanes/AcOEt) to provide the desired product in 78% yield (from **5**) as a dark orange solid. ^1^H NMR (400 MHz, Acetone-d^6^) *δ* (ppm): 1.11 (t, 3H, *J* = 7.0 Hz, CH_3_), 1.60 (s, 1H, OH), 3.48 (t, 2H, *J* = 7.2 Hz, N-CH_2_), 3.51 (q, 2H, *J* = 7.1 Hz, CH_2_), 3.81 (t, 2H, *J* = 6.0 Hz, CH_2_-OH), 6.61 (d, 2H, *J* = 8.8 Hz, CH-Ar-N), 7.35 (d, 2H, *J* = 8.4 Hz, N-Ar-C≡C-CH), 7.63 (d, 2H, *J* = 9.2 Hz, CH-Ar-C≡C-NO_2_), 8.19 (d, 2H, *J* = 8.8 Hz, CH-Ar-NO_2_). ^13^C NMR (100 MHz, Acetone-d^6^) *δ* (ppm): 12.2 (CH_3_), 45.5 (N-CH_2_), 52.7 (CH_2_), 60.8 (CH_2_-OH), 72.1 (C≡C-C≡C), 79.2 (C≡C-C≡C), 111.4 (N-Ar-C-C≡C), 112.3 (CH-Ar-N), 124.1 (CH-Ar-NO_2_), 129.9 (NO_2_-Ar-C-C≡C), 133.3 (NO_2_-Ar-CH-C≡C), 134.7 (N-Ar-CH-C≡C), 147.8 (C-NO_2_), 149.9 (C-N). FT-IR, *υ_max_* (cm^−1^): 3100, 1597, 848, 814 (Ar-H, Ar-C), 3412, 1071 (O-H, C-OH), 2952, 1458 (CH_3_), 2922, 1400 (CH_2_), 2201, 2135 (N-C≡C, C≡C- NO_2_), 1510, 1349 (Ar-NO_2_). UV-Vis, *λ_max_* = 424 nm. mp = 168–170 °C.

#### 3.2.5. 2.,5-bis(prop-2-ynyloxy)benzoyl chloride (**8**)

Was obtained a white compound can be easily hydrolyzed, so that it was used without any further purification in the subsequent reaction with a yield: 97%.

#### 3.2.6. 2-(ethyl(4-((4-nitrophenyl)buta-1,3-diynyl)phenyl)amino)ethyl-2,5-*bis*(but-2-ynyloxy)benzoate (**9**)

The product was purified by column chromatography on silica gel (1:1 hexane/AcOEt, added 5% of MeOH) to afford the desired product in a 91% yield (from **7**) as an orange powder. ^1^H NMR (400 MHz, CDCl_3_) *δ* (ppm): 1.27 (t, 3H, *J* = 8 Hz, CH_3_), 2.50 (t, 1H, *J* = 4 Hz, C≡H), 2.53 (t, 1H, J = 4 Hz, C≡H), 3.60 (q, 2H, *J* = 8 Hz, CH_2_), 3.82 (t, 2H, *J* = 8 Hz, N-CH_2_), 4.52 (t, 2H, *J* = 8 Hz, COO-CH_2_), 4.63 (d, 2H, *J* = 4 Hz, O-CH_2_), 4.73 (d, 2H, *J* = 4 Hz, O-CH_2_), 6.83–6.87 (m, 2H, CH-Ar-N), 7.08-7.15 (m, 1H, CH-Ar-CO), 7.39 (d, 2H, *J* = 4 Hz, CH-Ar-*ortho*-COO), 7.89–7.94 (m, 4H, C≡C-CH-Ar-NO_2_), 8.30–8.34 (m, 2H, CH-Ar-NO_2_). ^13^C NMR (100 MHz, Acetone-d^6^) *δ* (ppm): 13.2 (CH_3_), 42.8 (N-CH_2_), 45.6 (CH_2_), 57.4 (O-CH_2_), 59.1 (O-CH_2_), 66.6(CH_2_-COO), 73.9 (C≡C-C≡C), 76.5 (CH), 77.1 (C≡C-C≡C), 79.9 (C≡CH), 113.0 (N-Ar-C-C≡C), 115.6 (CH-Ar-N), 116.2 (C-Ar-COO), 116.9 (CH-Ar-*ortho*-COO), 121.1 (CH-Ar-*meta*-COO), 123.2 (CH-Ar-*para*-COO), 124.7 (CH-Ar-NO_2_), 131.2 (NO_2_-Ar-C-C≡C), 134.1 (N-Ar-CH-C≡C), 135.2 (NO_2_-Ar-CH-C≡C), 146.6 (C-NO_2_), 147.3 (C-N), 150.7 (C-O-*ortho*-COO), 152.0 (C-O-*meta*-COO), 163.4 (COO). FT-IR, *υ_max_* (cm^−1^): 3100, 1591, 854, 813 (Ar-H, Ar-C), 2960, 1456 (CH_3_), 2926, 1493 (CH_2_), 3289, 2121(C≡H), 2199(C≡C), 1719, 1196 (Ar-COO), 1518, 1340 (Ar-NO_2_) 1282, 1074, 1032 (Ar-CO). UV-Vis *λ_max_* = 423 nm. (FAB^+^) (*m*/*z*); 546.1785. mp = 175 °C.

#### 3.2.7. poly(2-(ethyl(4-((4-nitrophenyl)buta-1,3-diynyl)phenyl)amino)ethyl2,5-bis(but-2-ynyloxy) benzoate) (**10**)

The polymerization procedure was performed according to the oxidative coupling polymerization process (Hay Reaction) [45]. The substance was obtained as dark-orange polymer. ^1^H NMR (400 MHz, TFA-*d*) *δ* (ppm): 1.26 (s, 3H, CH_3_), 3.18–3.27 (m, 3H), 3.48–3.54 (m, 2H), 3.78–4.76 (m, 12H), 7.24–8.16 (m, 11H). ^13^C NMR (100 MHz, DMSO-*d*_6_) *δ* (ppm): 11.6, 38.1, 42.1, 42.4, 44.5, 47.8, 56.3, 57.5, 58.9, 62.3, 64.6, 66.4, 70.0, 71.6, 75.8, 79.4, 87.0, 104.9, 111.1, 111.5, 113.7, 114.3, 116.7, 116.8, 119.8, 120.0, 123.5, 123.8, 127.7, 130.1, 132.0, 133.1, 134.1, 147.0, 148.6, 150.7, 151.0, 151.1, 151.3, 164.3, 164.8, 166.2. FT-IR, *υ_max_* (cm^−1^): 3100, 1599.73, 853.38, 813.36 (Ar-H, Ar-C), 2963.7, 1456.05 (CH_3_), 2930, 1491 (CH_2_), 2197 (C≡C), 1719, 1194 (Ar-COO), 1518, 1339 (Ar-NO_2_), 1280, 1075, 1024 (Ar-CO). UV-Vis *λ_max_* = 430 nm. *Mw* = 12560; *Mw*/*Mn* = 1.548.

### 3.3. Thin Film Deposition and Corona Poling Process

Organic thin films were deposited onto ITO-coated glass substrates via spin coating procedures, implementing the obtained polymeric product (using NMP-based solutions at 10 wt %). Film depositions were carried out at 120 °C, and the obtained films were vacuum dried at ~80 °C for ~1 h in order to remove residual NMP-solvent. The electrical molecular alignment was performed implementing an iron needle-point cathode placed at a needle-film gap distance of ~1.4 cm. The applied DC-voltage was in the range of 8–9 kV. Polymer film samples were mounted on a temperature-controlled heating plate to start the poling process from 60 °C to temperatures just above the *T_g_*-values (heating rate: 90 °C h^−1^). The poling process was maintained for 30 min (the sample temperature was fixed at *T_g_* + 10 °C). The poled films were then cooled to 60 °C (DC-field state: ON) and then to room conditions (*T* ≈ 25 °C, DC-field state: OFF). Afterwards, the film samples were characterized by UV-Vis spectroscopic measurements in order to determine the corresponding order parameter.

### 3.4. Morphological AFM-Film Inspections

AFM-surface analyses of deposited films were performed on poled and unpoled film samples to determine their structural morphology using a Park-AutoProbe CP apparatus. The acquisition of images was carried out in non-contact mode with an applied interaction force between the sample and the AFM-tip of 1.5 nN. The AFM system was equipped with a SiN sharpened MIcrolever^TM^ tip with typical force constant of 0.05 N and resonant frequency of 22 KHz, which specify the mechanical characteristics of the cantilever (typical constants of the instrument).

### 3.5. NLO/SHG Measurements

Electrically poled organic films were tested as active media for quadratic *χ*^(2)^-NLO/SHG effects. The experimental set-up comprises a commercial Q-switched Nd:YAG-Laser system (Surelite II-Continuum/Gaussian mode: TEM_00_, *λ_ω_* = 1064 nm, rep. rate = 10 Hz and pulse width: *τ* < 22 ns) used to provide the fundamental excitation. The pulse power was set to ~80 J, and the laser beam was focused by means of an *f* = 5 mm focusing lens, so that peak irradiances on the order of ~8 MW/cm^2^ were achieved at the focal spot on the sample. This value was below the damage threshold supported by the samples. The polarization of the fundamental beam (parallel/P or perpendicular/S polarizing geometry) was selected by means of an IR-coated Glan-Laser polarizer and a *λ*/2-retarder plate. A second polarizer used as an analyzer allowed the characterization of the SHG-signals. The SHG-waves (*λ_2ω_* = 532 nm) were detected using a photomultiplier tube (PMT) placed after convenient interferential optical filters (532 ± 5 nm). The SHG experimental set-up was calibrated by means of an *α*-quartz crystal wedged along the *d*_11_^(2)^-direction (*d*_11_^(2)^ ≈ 0.64 pm V^−1^ = 0.5 *χ*_11_^(2)^), which is frequently used as the NLO-reference standard via the Maker fringes method [33,34,35]. NLO-signals captured from photo-detectors were recorded with a digital oscilloscope and acquired via a LabView master program.

### 3.6. EPR Spectroscopy

EPR spectra at room temperature (295 K) were measured under non-saturating conditions of microwave power on a Bruker Elexys E500 spectrometer, at approximately 9.86 GHz (X-band) and 100 KHz modulation. The EPR spectra were evaluated and simulated using the easyspin ver. 5.1, and g-values were calculated by measuring accurately the magnetic field and the microwave frequency. For the interpretation of the EPR spectra, we use the usual spin Hamiltonian for Cu(II), S = 1/2, specie.

$${\hat{H}}_{S}=\left(g_{\mathrm{Perp}}+g_{\mathrm{paral}}\right)\beta \mathrm{BS}+H_{F,\mathrm{Cu}}+H_{F,N}$$

where g_Paral_, and g_Perp_ are the parallel and perpendicular components of the g-tensors of one Cu(II) center, respectively. The term H_F,Cu_ is the hyperfine contribution from the copper nucleus, I(Cu) = 3/2, and H_F,N_ is the hyperfine contribution from the nitrogen nucleus, I(N) = 1.

## 4. Conclusions

The synthesis and characterization of polymer **10** was achieved alongside the determination of quadratic NLO/SHG-measurements. The thermal properties, amorphous nature and good solubility of this polymer allowed the fabrication of high quality spin-coated optical films with a stable mechanical structure. The samples analyzed by this methodology exhibited low absorption within the visible spectral range, and the corresponding poled samples displayed outstanding SHG-signals. In fact, an efficient and stable chromophore alignment, together with an inherent rigidity of the polymer main chain, can support the final molecular configuration, preserving the polar diacetylene chromophore orientation. In agreement to the Maker fringes technique, this polymeric material exhibits an intense and stable SHG-response in off-resonant experimental conditions, evidencing low dipolar aggregate interactions, which enhances the NLO/SHG-response. The obtained NLO-coefficients are larger than those reported for common polymers containing *azo*-compounds and tolan-units as NLO-chromophores. 

This behavior makes our push-pull polymer a promising material for NLO photonic/opto-electronic applications. The polymer we present in our work exhibits attractive mechanical and opto-electronic properties, making it a potential candidate for technological devices [46]. Finally, based on a thermogravimetric analysis, it is expected that the polymeric structure could be cross-linkable by the thermal cross-polymerization of the diacetylene groups, allowing enhanced mechanical and NLO-properties.

## Figures and Tables

**Table 1:** Comparison of the non-linear optical (NLO)-coefficients of polymer **10** with other polymers.

Compound	*χ*_33_^(2)^ = *χ_zzz_*^(2)^/pm V^−1^	Ref.
**10**	280	
**11**	186.8	[29]
**12**	70	[44]
**13**	119	[43]
